# Tracking the neuroplastic changes associated with transcranial direct current stimulation: a push for multimodal imaging

**DOI:** 10.3389/fnhum.2013.00495

**Published:** 2013-08-27

**Authors:** Michael A. Hunter, Brian A. Coffman, Michael C. Trumbo, Vincent P. Clark

**Affiliations:** ^1^Psychology Clinical Neuroscience Center, The University of New MexicoAlbuquerque, NM, USA; ^2^Department of Psychology, The University of New MexicoAlbuquerque, NM, USA; ^3^Department of Psychiatry, The University of New Mexico School of MedicineAlbuquerque, NM, USA; ^4^The Mind Research Network and Lovelace Biomedical and Environmental Research InstituteAlbuquerque, NM, USA; ^5^Psychiatry Research, New Mexico Raymond G. Murphy VA Healthcare SystemAlbuquerque, NM, USA; ^6^Department of Neurosciences, The University of New Mexico School of MedicineAlbuquerque, NM, USA

**Keywords:** Multimodal Imaging, transcranial direct current stimulation (tDCS), neuroplasticity, magnetic resonance spectroscopy (MRS), functional magnetic resonance imaging (fMRI), electroencephalography (EEG)

## Introduction

The human brain operates through an intricate balance of *excitatory* and *inhibitory* processes. Transcranial direct current stimulation (tDCS) is a non-invasive technique that is generally assumed to work by increasing the level of brain activity near the anode (positive polarity), while decreasing it near the cathode (negative polarity). However, this is based in part on untested assumptions: the exact (cellular and synaptic) inhibitory or excitatory processes that are targeted preferentially using either polarity is still an open area of research. Furthermore, the relationship between electrode polarity and membrane excitability is highly contingent upon stimulation parameters (e.g., montage, intensity, cognitive task, etc.). Although neuroimaging has been utilized to verify these general effects in the brain, further development is needed to advance our understanding of the mechanisms by which tDCS produces changes across different levels of the nervous system. To date, tDCS has produced reliable changes in neurometabolite concentration using magnetic resonance spectroscopy (MRS; Rango et al., 2008; Stagg et al., 2009; Clark et al., 2011); whole-brain functional connectivity using functional magnetic resonance imaging (fMRI; Baudewig et al., 2001; Kwon et al., 2008; Polanía et al., 2011a, 2012; Peña-Gómez et al., 2012; Sehm et al., 2012, 2013; Park et al., 2013; see Turi et al., 2012 for review); and neural oscillations and event-related potentials using electroencephalography (EEG; Keeser et al., 2011; Polanía et al., 2011b; Jacobson et al., 2012) or magnetocenphalography (MEG; Venkatakrishnan et al., 2011). While each of these imaging techniques provides information at specific levels within the brain's neural architecture, from the micro-scales (e.g., neuro-metabolites) to the macro-scales (e.g., population-level neural synchronization), no study has combined more than one imaging modality with tDCS in order to track neuroplastic changes across these different scales.

In this Opinion Article, we briefly summarize the progress made on tracking tDCS-induced neuroplastic changes using single imaging modalities (specifically MRS, fMRI, and EEG). We then demonstrate the need for multimodal imaging, with the goal of establishing a more comprehensive examination of both local and global neuroplastic changes due to tDCS. Such a design would enable measurements of brain chemistry and large-scale functional connectivity within the same subject and tDCS session, thus capturing interactions of these measures that may account for significant variability in cognition and behavior.

## Neurochemical markers of neural plasticity

Given that anodal tDCS leads to lasting changes in behaviors related to learning and memory (Brasil-Neto, 2012; Clark et al., 2012). It is hypothesized that its effects may interact with long-term synaptic potentiation (LTP) through changes in specific neurotransmitter levels. Proton magnetic resonance spectroscopy (^1^H-MRS) allows for accurate quantification of certain neurotransmitters within a localized region of the brain. To date, there have been few published MRS-tDCS studies where data were acquired immediately before and after tDCS. For instance, Rango et al. (2008) demonstrated that anodal stimulation over right M1 resulted in increased myoinositol concentration beneath the stimulating electrode. Given that myoinositol is linked to membrane phospholipid metabolism and is associated with the LTP second messenger system (Rango et al., 2008), this supports the hypothesis that tDCS operates in part via an LTP-like mechanism. Along these lines, the NMDA antagonist dextromethorphane has been shown to prevent lasting effects of tDCS on motor-evoked potentials (MEPs), suggesting that the mechanisms affected by tDCS may be dependent on the NMDA glutamate receptor subtype (Liebetanz et al., 2002; Nitsche et al., 2003).

Further research using ^1^H-MRS has demonstrated an increase in Glx (combined glutamate and glutamine) and NAA (N acetyl aspartate) under the anodal electrode (located near P4), compared to the opposite hemisphere (Clark et al., 2011), further supporting the hypothesis of increased metabolism and increased glutamatergic activity interacting with LTP mechanisms. Stagg et al. (2009) found that GABAergic and glutamatergic activity was increased after anodal stimulation (over left M1) and was reduced after cathodal stimulation. Moreover, changes in GABA concentration were inversely related to the amount of motor learning. Furthermore, Fritsch et al. (2010) found that genetic polymorphisms in the gene that codes for brain-derived neurotropic factor (BDNF) mediate the neuroplastic effects of direct current stimulation: Val66Val polymorphism of the BDNF gene was found to be beneficial to motor skill learning through training in humans, while Met66Met knockin mice showed decreased effects of local felid stimulation in M1 slices in mice.

While results differ to some degree under various stimulation and MRS parameters, ^1^H-MRS has proven to be a useful tool for the assessment of the neurochemical changes due to tDCS. Together, the observed effects of stimulation are consistent with the modulation of LTP and/or LTD mechanisms, with changes in myoinositol, Glx, GABA, NAA, and BDNF consistent with LTP-type processes.

## TDCS-induced changes in brain dynamics: large-scale functional connectivity

Functional connectivity is a statistical measure of the relationship between multiple brain regions. This measure provides information about whole-brain information integration, and can predict individual variability in cognitive performance in both healthy controls and patients (Friston, 1994; Bullmore and Sporns, 2009). TDCS may modify the threshold for LTP and LTD within and across structurally connected brain regions that comprise a functional network (Venkatakrishnan and Sandrini, 2012). Indeed, previous studies have shown that tDCS produces alterations across widespread distributed brain networks that extend far from the area of stimulation (Lang et al., 2005; Roche et al., 2011; Polanía et al., 2012).

Co-variation of resting-state fluctuations in blood oxygen level-dependent (BOLD) fMRI have been interpreted as measures of the *intrinsic* functional connectivity within the brain (Raichle et al., 2001; Fox et al., 2005). A recent investigation of the dynamic interactions within and across intrinsic resting-state networks before and after the application of anodal tDCS over the dorsal lateral prefrontal cortex (DLPFC; cathode over contralateral supraorbital area) revealed a redistribution of activity across resting-state networks (Peña-Gómez et al., 2012). Active tDCS resulted in site-specific increases in *synchronous* activity between lateral frontal and parietal areas and *asynchronous* activity between brain regions comprising the default-mode network (i.e., medial prefrontal and medial posterior areas; Peña-Gómez et al., 2012). In related work, a graph theory analysis of resting-state fMRI data found that anodal stimulation over left M1 combined with cathodal stimulation over the contralateral frontopolar cortex resulted in a global *decrease* in the long-distance topological functional coupling of the left M1 with the rest of the brain (Polanía et al., 2011a). It was hypothesized that the local increase of spontaneous activity due to anodal stimulation over M1 may have decreased the neuronal signal-to-noise ratio and consequently decreased the synchronization with other brain regions.

Other fMRI studies have investigated network connectivity changes induced by tDCS (e.g., Polanía et al., 2012; Sehm et al., 2012, 2013; Park et al., 2013), with all of them suggesting that tDCS over important network hubs (including DLPFC and M1) both increases local spontaneous activity and modulates functional connectivity across brain regions. Thus, network-level changes associated with tDCS may allow for the characterization of neuroplastic changes at the level of whole-brain functional connectivity.

## Cortical oscillations and event-related potentials

EEG has provided information about changes in neural oscillations associated with tDCS. For instance, Keeser et al. (2011) found that anodal stimulation over left DLPFC with the cathode over right frontopolar cortex decreased delta power and marginally increased beta power. Likewise, Jacobson et al. (2012) demonstrated a selective reduction in theta-band power following anodal stimulation over the right inferior frontal gyrus (cathodal stimulation over left orbitofrontal cortex) compared to sham.

Together, these findings are consistent with the hypothesis that anodal stimulation leads to a shift from lower to higher oscillatory frequencies (Keeser et al., 2011). Indeed, it is possible that tDCS differentially modulates cortical oscillatory frequencies, and may potentially influence mental states and behavioral performance. However, it is important to note that there is limited mechanistic evidence (from both animal and human studies) that can explain the effects of direct current stimulation on the shift from higher to lower frequency oscillations in humans. Nonetheless, Fröhlich and McCormick (2010) showed that weak constant and sine-wave electric fields enhance and entrain slow oscillations, which was hypothesized to represent dynamic feedback mechanisms that modulate and guide network-wide synchronization at different frequency bands. To this end, electric fields may have functional implications on the interplay between different cortical areas, local processing and oscillations at specific frequencies. Additionally, using a computational network model, (Reato et al., 2010) showed that incremental polarization by weak currents lead to a small increase in firing rate, with excitatory spike times broadly distributed across the theta cycle, resembling *in vivo* recordings of theta-modulated gamma activity. Altogether, EEG measures provide precise information on the timing of brain activity, allowing for a temporally-accurate account of tDCS alterations of brain dynamics, which may elucidate the mechanisms by which tDCS operates.

## Combining markers of the mechanisms affected by tDCS

When taken together, the neuroimaging methods reviewed so far provide a complex spatiotemporal description of the functional effects of tDCS and depict changes in brain function resulting from stimulation. However, stimulation protocols (e.g., duration, intensity, cognitive paradigm, electrode size and montage) vary across studies and therefore introduce another layer of complexity when trying to compare and interpret results from multiple studies. These parameters can have complex effects on experimental results—for instance, increasing stimulation intensity can have non-linear effects on motor evoked-potential amplitudes (Batsikadze et al., 2013). Additionally, differences in individual subject characteristics (e.g., sex, handedness, age, etc), and other factors may lead to discrepancies between studies. It is proposed that these imaging methods must be combined in a more coordinated way in order to better identify and characterize markers of neuroplasticity induced by tDCS. For instance, a combined MRS-fMRI-EEG study using the same participants and tDCS protocol could utilize measures of specific neurometabolites (e.g., Glx, GABA, BDNF and myoinositol) to be compared with network-level functional connectivity with fMRI and changes in frequency bands (e.g., delta, theta and gamma) with EEG. While still imperfect, and constrained by the limits of each method, a combination of neuroimaging methods could still provide a deeper understanding of the mechanisms by which tDCS influences brain function and behavior.

A recently published study demonstrated the feasibility of combined EEG-fMRI with transcranial magnetic stimulation (Peters et al., 2013), which suggests that such a protocol could be implemented with a tDCS unit and an added MRS sequence. Furthermore, multi-site collaborations, meta-analyses and replication studies can be fostered with standardized stimulation protocols (e.g., size of electrode sponges, duration and intensity of stimulation) and image acquisitions.

In summary, it has been shown that tDCS is well-poised as a novel intervention to alter learning and memory (Clark et al., 2012), attention (Coffman et al., 2012), and a variety of other cognitive functions in healthy and clinical populations (see Kuo et al., 2013 and Floel, 2013 for reviews). Understanding the mechanisms by which these changes occur could help to increase its effectiveness and help us to understand the neural architecture and dynamics of the human brain. For instance, increasing both the glutamatergic transmission and functional connectivity that is otherwise impaired in schizophrenia (Friston, 1998; Szulc et al., 2013) could lead to effective therapies that combine both pharmacology and stimulation (Brunelin et al., 2012) protocols. Thus, the importance of investigating potential interactions across these levels of analyses could inform future hypotheses for the most optimal cortical targets and specific methods of brain stimulation for neurological and psychiatric disorders. With continued multimodal imaging work akin to that conducted by Peters et al. (2013), we can further our understanding of the neuroplastic effects of tDCS that will ultimately translate to clinical applications, resulting in more effective and well-controlled therapeutic interventions.
